# A Person-Centered Analysis of Adolescent Multicultural Socialization Niches and Academic Functioning

**DOI:** 10.1007/s10964-023-01828-0

**Published:** 2023-07-26

**Authors:** Maciel M. Hernández, M. Dalal Safa, Olga Kornienko, Adam A. Rogers, Thao Ha

**Affiliations:** 1 Department of Human Ecology, University of California, Davis, CA USA; 2 Department of Psychology and Neuroscience, University of North Carolina at Chapel Hill, Chapel Hill, NC USA; 3 Department of Psychology, George Mason University, Fairfax, VA USA; 4 School of Family Life, Brigham Young University, Provo, UT USA; 5 Department of Psychology, Arizona State University, Tempe, AZ USA; 6 REACH Institute, Department of Psychology, Arizona State University, Tempe, AZ USA

**Keywords:** Academic engagement, Academic expectations, Academic functioning, Adolescents, Latent profile analysis, Multicultural socialization

## Abstract

Despite the growing cultural diversity worldwide, there is scarce research on how socialization processes prepare youth to respond to increasing multicultural demands and the degree to which these socialization opportunities inform youth academic functioning. This study used a person-centered approach to identify profiles or niches based on the degree and consistency of multicultural socialization experiences across school, peer, and family settings and to examine the associations between identified niches and markers of academic functioning (i.e., emotional and behavioral academic engagement, academic aspirations and expectations) in a sample of adolescents (*N* = 717; *M*_age_ = 13.73 years). Participants (49.9% girls) were from the U.S. Southwest and represented multiple ethno-racial backgrounds (31.8% Hispanic/Latinx, 31.5% Multiethnic, 25.7% White, 7.3% Black or African American, 1.4% Asian American or Pacific Islander, 1.4% American Indian or Alaska Native, and 1% Arab, Middle Eastern, or North African). Six distinct multicultural socialization niches were identified. Three niches had similar patterns across school-peer-family but ranged in the degree of socialization. The *cross-setting similar higher socialization* niche (Niche 6) demonstrated greater socialization than the *cross-setting similar moderate* (Niche 5) and *lower socialization* (Niche 4) niches, which had moderate and lower socialization, respectively. Three niches demonstrated cross-setting dissimilarity which ranged in the type of cross-setting contrast and the degree of socialization. The *cross-setting dissimilar school contrast socialization* niche (Niche 3) had greater dissimilarities between socialization opportunities in the school setting compared to the peer and family settings and demonstrated the lowest levels of socialization of all niches. The other two niches, the *cross-setting dissimilar peer contrast* (Niche 1) and *greater peer contrast socialization* (Niche 2) niches had larger dissimilarities between socialization opportunities in the peer setting than the school and family settings. In the former, however, the contrast was lower, and socialization ranged between very low to low. In the latter, the contrast was higher and socialization ranged from very low to moderate. Most adolescents were in the *cross-setting similar lower socialization* niche or in the *cross-setting dissimilar* niches. Adolescents in the *cross-setting similar higher multicultural socialization* demonstrated greater emotional and behavioral academic engagement than adolescents in most of the other niches. Adolescents in the *cross-setting dissimilar school contrast* niches demonstrated lower emotional and behavioral academic engagement and lower academic expectations than adolescents in some of the other niches. The results emphasize the collective role of school, peer, and family multicultural socialization on emotional and behavioral academic engagement.

## Introduction

We live in a multicultural world defined by cultural diversity. Indeed, the United States (U.S.) is more ethnically and racially diverse than ever (U.S. Census Bureau, 2021, August 12), and similar growth has emerged worldwide (Pew Research Center, 2019, April 22); thus, youth interact more frequently with peers from multiple ethno-racial backgrounds (Nishina et al., 2019). These changing demographics have essential implications for the development and adjustment of youth from ethno-racial majoritized and minoritized groups (Berry et al., 2022), particularly for their academic adjustment as youth attend schools with growing ethno-racial diversity. There is scarce research on how socialization processes equip youth to respond to increasing multicultural demands and the degree to which these socialization experiences may inform youth academic functioning (i.e., academic engagement, aspirations, and expectations). This study addresses this gap by examining intercultural or multicultural socialization experiences across multiple settings (i.e., schools, peers, and families) and their links with youth academic functioning.

*Intercultural or multicultural socialization* involves efforts to teach youth about *cultural pluralism* and the importance of *equal treatment* across members from all ethno-racial groups (Berry & Sam, 2014). *Multicultural socialization* is theorized to be a critical process supporting youth academic functioning in multicultural societies (Barrett, 2018). Through multicultural socialization, salient proximal settings such as schools, peers, and families provide youth with opportunities to learn about multiple cultures, appreciate the value of cultural pluralism, and practice multicultural competencies (Berry et al., 2022). These opportunities and competencies are theorized to support youth’s overall adjustment in multicultural societies (Barrett, 2018), particularly their academic functioning (Nishina et al., 2019).

Multiculturalism research at the individual and societal levels has substantially increased in the past decade (e.g., The Oxford Handbook of Multicultural Identity). This body of work points to the benefits and challenges youth experience in multicultural societies and notes variability across proximal settings (Benet-Martínez & Hong, 2014). Although limited, recent empirical work has focused on multicultural socialization in the school setting and provides evidence for the positive link between multicultural socialization and youth academic functioning (e.g., Byrd, 2019). However, multicultural socialization beyond the school setting, including peer and family settings, and how variability across these intersecting socialization settings informs youth academic functioning is unknown.

Guided by ecological models highlighting the role of intersecting forces across proximal settings informing youth’s adjustment (e.g., Bronfenbrenner & Morris, 2006), the current study (1) identifies *multicultural socialization niches* defined by the opportunities afforded to youth to learn about cultural pluralism and the importance of equal treatment of all ethno-racial groups across *school, peer, and family settings*, and (2) examines how these niches inform adolescent academic functioning in a U.S. ethno-racially diverse sample. This study focuses on *academic functioning*—marked by emotional and behavioral academic engagement, academic aspirations, and academic expectations (Skinner et al., 2022)—because this is a multifaceted and salient developmental task that significantly decreases throughout adolescence (Eccles & Roeser, 2011) but has significant implications for future career (May & Witherspoon, 2019) and academic success (Wang & Peck, 2013).

### Multicultural Socialization Niches

There is strong theoretical justification for considering the cross-setting, unique (person-centered) nature of adolescent multicultural socialization niches and how these unique niches inform adolescent academic functioning (e.g., White et al., 2018). During adolescence, socialization settings outside the family become increasingly salient (Crosnoe & Benner, 2015), particularly school and peer settings become prominent (Eccles & Roeser, 2011). Importantly, socialization processes are influenced by the beliefs and practices that characterize these settings (Super & Harkness, 2002), emerge from adaptive cultural models reflecting individual and societal values (White et al., 2018), and involve multidirectional, interactive processes between youth and their settings (Umaña-Taylor et al., 2013). It follows that cross-setting variability and intersecting forces shape the multicultural socialization niches which adolescents are negotiating (Super & Harkness, 2002), and these unique niches inform youth attitudes toward cultural diversity (Miklikowska et al., 2019), the development of multicultural competencies (White et al., 2018), and ultimately their academic functioning (e.g., García Coll et al., 1996).

Prior empirical work provides evidence of the unique and intersecting nature of youth cultural socialization niches. For instance, research assessing a combination of heritage (i.e., efforts to teach youth about their heritage and cultural background); national (i.e., efforts to teach youth about U.S. mainstream culture); and multicultural socialization identified multiple socialization niches with different *degree* (higher vs. lower levels) and *consistency* (similar vs. dissimilar) of socialization experiences across school and family settings (Byrd & Ahn, 2020). Similarly, research examining heritage and national cultural socialization separately also identified multiple socialization niches which varied in the degree and consistency of socialization experiences across peer and family settings (Wang & Benner, 2016). This work highlights the considerable heterogeneity in U.S. adolescent experiences of cultural socialization across school, peer, and family settings and the importance of using cross-setting, person-centered approaches to capture this variability (i.e., degree and consistency) regarding multicultural socialization.

Considering the variability in the *degree* of multicultural socialization experiences, some adolescents may be embedded in or negotiating higher multicultural socialization niches where they are frequently provided opportunities to learn about other cultures across these settings. In contrast, others may be part of lower multicultural socialization niches with little to no opportunity. Consistent with prior theoretical (Super & Harkness, 2002) and empirical work (Umaña‐Taylor & Hill, 2020) highlighting the importance of frequent, ample socialization opportunities for youth to learn from and draw adjustment-related benefits from these experiences, it is likely that youth negotiating niches with higher multicultural socialization demonstrate more knowledge and awareness about other cultures and thus gain more academic-related benefits.

Importantly, beyond variability in the degree of multicultural socialization, variability can also emerge in the *consistency* (similarity vs. dissimilarity) of socialization messages and opportunities that youth experience across their proximal settings (Byrd & Ahn, 2020). Specifically, adolescents may negotiate niches where schools, peers, and families match in the content and degree of socialization efforts (*cross-setting similarity*) or in niches where there is a mismatch across these settings (*cross-setting dissimilarity*). Prior research underscores the importance of cross-setting similarity in youth cultural socialization experiences for their academic functioning (Wang & Benner, 2016). These studies, however, have not explicitly focused on multicultural socialization.

Findings from prior studies suggest that variability in both degree and consistency of cultural socialization experiences is important. Further, ecological models (e.g., García Coll et al., 1996) emphasize that school, peer, and family settings influence the kinds of transactions adolescents negotiate and that mutually reinforcing repetition of similar influences across these settings has important implications for youth adjustment (Super & Harkness, 2002). Thus, in multicultural socialization niches characterized by cross-setting similarity, adolescents may encounter comparable cross-setting messages and opportunities to learn about other cultures and are likely to experience more academic-related benefits from these socialization experiences in a cohesive niche. Conversely, given that socialization efforts reflect adaptive cultural models (White et al., 2018), adolescents who experience cross-setting dissimilarity may be exposed to competing affordances and demands in each of these settings, and this mismatch may likely diminish their understanding of the value of cultural diversity and thus may have a cost to their academic functioning.

A cross-setting, person-centered view of the adolescent niche may be particularly important for the current examination because multicultural socialization processes involve a degree of understanding that cultural diversity permeates all aspects of adolescents’ lives in multicultural societies (Benet-Martínez & Hong, 2014). Further, adolescent academic functioning within schools ranging in ethno-racial diversity involves the ability to interact with and learn from individuals from diverse cultures, ethnicities, and races; to develop a sense of belonging amid cultural pluralism (Barrett, 2018); and to meet multiple demands, which may be, at times, competing with one another (Celeste et al., 2019).

### Links Between Multicultural Socialization Niches Across Schools, Peers, and Families and Youth Adjustment

Multicultural socialization opportunities have been theorized to support youth overall development and adjustment (Barrett, 2018), including their academic functioning (Nishina et al., 2019). Further, empirical work supports these notions. Given the scarce literature focused on the link between multicultural socialization and youth academic functioning, this study draws from work capturing related types of cultural socialization experiences and links with different indicators of psychosocial adjustment. Most empirical work on multicultural socialization has focused on the school setting and provides support for the positive link between multicultural socialization and academic functioning. For instance, multicultural socialization has been associated with greater school belonging and with greater college satisfaction among a U.S. ethno-racially diverse sample of college students (Byrd, 2019). Further, in German adolescent samples, multicultural socialization has been directly (Schachner et al., 2021) and indirectly via youth heritage and national identities (Schachner et al., 2016) associated with positive psychosocial adjustment, including academic functioning. These studies highlight how multicultural socialization in schools, likely through teachers’ efforts to promote positive intergroup contact (Karataş et al., 2023) and responses to ethnic-racial victimization (Bayram Özdemir & Özdemir, 2020), inform different indicators of academic functioning but do not consider the intersecting role of peer and family socialization settings.

During adolescence, peers become an important socialization setting providing youth opportunities to learn about themselves and others through various cultural socialization experiences (Eccles & Roeser, 2011). Ethnographic research reveals that adolescents and young adults engage in meaningful conversations about their heritage or ethnic-racial identity development, racial inequality, and discrimination with their friends and peers (Moffitt & Syed, 2021; Syed & Juan, 2012). Further, peers play a role in the transmission of culture and in youth exploration and navigation of what it means to be a member of a particular ethnic, racial, or cultural group (Wang & Lin, 2023), as well as in the promotion of openness to cultural diversity and intergroup peer inclusion (Burkholder et al., 2021; Killen et al., 2022). Social network-informed studies provide additional evidence for peers playing a vital role in cultural socialization. This work shows that adolescents from the U.S and Northern Europe influence each other to become similar in terms of their attitudes toward intergroup relationships (Zingora et al., 2020) and anti-immigrant and xenophobic attitudes (Bohman & Kudrnáč, 2022; van Zalk & Kerr, 2014). Prior research underscores that friends and peers contribute to cultural socialization by shaping heritage or ethnic-racial (Rivas-Drake et al., 2017; Santos et al., 2017) and national identity development (Umaña-Taylor et al., 2020), and these have important implications for youth academic functioning (Safa et al., 2022). These studies elucidate the important socialization roles of friends and peers in adolescent social and academic development but have not focused on multicultural socialization efforts.

Given the increasingly salient role of peers as a primary socialization setting, this study theorizes that peer multicultural socialization would foster adolescent academic functioning. Specifically, peer efforts to support adolescents' understanding of cultures and ethnic-racial groups other than one’s own and to foster positive intergroup contact may promote diversity and multicultural attitudes and skills, positive relationships with peers from different cultural backgrounds, and a sense of belonging in culturally plural academic settings (Nishina et al., 2019). These competencies are theorized to support adolescent academic functioning by providing youth with affective, behavioral, and cognitive tools to adequately respond to academic demands across multicultural settings (García Coll & Szalacha, 2004).

In the family setting, parents’ (or caregivers’) socialization processes aim to equip youth to thrive within specific (multi)culturally bounded contexts (Vélez-Agosto et al., 2017). Thus, parents participate in multiple socialization practices to achieve this goal (Bornstein & Lansford, 2010). For instance, parents engage in heritage culture socialization practices, and prior work has documented a positive link between youth’s opportunities to learn about their heritage background and their academic functioning (Huynh & Fuligni, 2008; Wang et al., 2020). Further, parents also engage in bicultural socialization processes that provide youth opportunities to learn about their heritage and the national culture (Cheah et al., 2013; Kim & Hou, 2016). These parental bicultural socialization experiences have been documented to be positively linked with adolescent psychosocial and cognitive adjustment (Knight et al., 2016; Zhang et al., 2018), but these studies did not focus on academic functioning.

Although prior work has not examined multicultural socialization, it follows that family multicultural socialization would foster adolescent academic functioning. Specifically, by providing youth with opportunities to develop an understanding of cultures and ethnic-racial groups other than one’s own, parents and caregivers help youth understand the challenges and opportunities of cultural pluralism and ethnic-racial socialization (for White children and youth, see Hazelbaker et al., 2022; for children and youth of color, see Rivas-Drake et al., 2022), internalize multiculturalism beliefs (Kim & Hou, 2016), and navigate everyday interactions with people from culturally diverse backgrounds (Neblett et al., 2012). These competencies may instill a greater sense of efficacy in navigating multicultural academic settings and demands (García Coll & Szalacha, 2004).

Schools, peers, and families are salient proximal settings comprising adolescent socialization niches (Super & Harkness, 2002). These settings work in tandem with one another, and their joint forces may promote or inhibit multicultural socialization goals and associated academic-related benefits. Adolescents in cross-setting similar niches with higher degrees of multicultural socialization are theorized to reap the most benefits regarding their academic functioning, and empirical work supports this notion. Indeed, youth negotiating niches characterized by cross-setting similarity with higher levels of peer and family heritage or national cultural socialization demonstrated greater academic adjustment than youth in other niches characterized by either cross-setting similarity with lower socialization levels or cross-setting dissimilarity (i.e., higher parent socialization; lower peer socialization; Wang & Benner, 2016). Similarly, adolescents negotiating niches characterized by cross-setting similarity with higher degrees of socialization (i.e., a combination of higher heritage, national, and multicultural socialization across school and family settings) demonstrated greater academic engagement and aspirations than adolescents in niches characterized by either cross-setting similarity with lower socialization levels or cross-setting dissimilarity (Byrd & Ahn, 2020). Across these studies, there was no difference between the lower-level cross-setting similar niche and the cross-setting dissimilar niche in academic outcomes, highlighting the significance of both degree and consistency of cultural socialization experiences across settings. These findings underscore the importance of youth exposure to at least moderate socialization opportunities and the need to consider the nuanced ways in which the degree and consistency of adolescent socialization niches inform their academic functioning and adjustment. The current study relies on a cross-setting, person-centered approach (Bergman, 2001) to capture the variability that characterizes adolescent multicultural socialization niches across school, peer, and family settings and to examine how these unique niches inform adolescent academic functioning.

### Indicators of Social Position and Multicultural Socialization Niches

Socialization efforts involve multidirectional, interactive processes between youth and their proximal settings (Umaña-Taylor et al., 2013). Gender, ethnicity or race, and parental nativity are key indicators of social position factors informing youth socialization processes and developmental pathways (Stein et al., 2016). Specifically, these factors may influence the affordances and demands youth encounter across school, peer, and family settings and their intersecting forces. In other words, these factors may inform the degree and consistency of multicultural socialization opportunities that youth experience across these settings.

Albeit limited, some research highlights the importance of considering the role of these social position indicators on youth cultural socialization niches. Specifically, prior work has documented that neither gender, ethnicity/race, or parental nativity (i.e., having at least one foreign-born parent) informed the types of heritage cultural socialization niches (peer and family settings) youth were negotiating (Wang & Benner, 2016). However, this research revealed that ethnicity/race and parental nativity informed youth’s national cultural socialization niches. Particularly, Latinx adolescents were more likely than Black adolescents to be in the cross-setting dissimilar national cultural socialization niche. Similarly, adolescents with at least one foreign-born parent were more likely to be in the cross-setting dissimilar national cultural socialization niche than those with only U.S.-born parents. No differences were observed for gender (Wang & Benner, 2016). Additionally, prior work focused on a combination of heritage, national, and multicultural socialization niches has documented that Black adolescents compared to White adolescents were less likely to be in the cross-setting dissimilar socialization niche (Byrd & Ahn, 2020). No differences were observed for gender (Byrd & Ahn, 2020). These findings point to the interplay between youth’s social position and cultural socialization experiences. Given the scarce research and that findings vary based on the type of socialization practices and indicators of social position, exploratory analyses were conducted to examine how gender, ethnicity/race, and parent nativity would inform the type of multicultural socialization niches youth are more likely to be negotiating.

## Current Study

Prior research has rarely examined an increasingly salient socialization process in multicultural societies, namely multicultural socialization, among a U.S. ethno-racially diverse adolescent sample. First, the current study identified adolescents’ multicultural socialization niches, defined by the degree and consistency of multicultural socialization experiences across school, peer, and family settings (Aim 1). Second, the study examined how these unique niches inform key markers of youth academic functioning (i.e., emotional and behavioral academic engagement; academic aspiration and expectations; Aim 2). Based on prior theoretical and empirical work, it was expected that several niches or profiles would emerge characterized by different types of degree and cross-setting consistency of multicultural socialization experiences (Hypothesis 1). Further, these unique niches were expected to have implications for youth academic functioning. Specifically, youth negotiating niches characterized by cross-setting similarity with higher levels of multicultural socialization were expected to demonstrate better academic functioning than youth in other niches (Hypothesis 2a). It was also expected that youth negotiating niches characterized by cross-setting dissimilarity with lower levels of multicultural socialization would demonstrate lower academic functioning than youth in other niches (Hypothesis 2b). Finally, based upon extant theory recognizing the influence of salient social position indicators (e.g., gender, ethnicity/race, and parental nativity) on youth socialization processes and developmental competencies, exploratory analyses examined whether these key indicators of social position informed the likelihood to be in a particular multicultural socialization niche (exploratory Aim 3).

## Methods

### Participants

Participants consisted of 717 students from two middle and two high schools from a public school district in the Southwestern U.S. Two-hundred eighty 6^th^ graders and 437 9^th^ graders participated in the study. Participants’ self-reported gender was 49.9% girls, 48.9% boys, and 1.1% other. Participants’ mean age was 13.73 years (*SD =* 1.54, range: 10–18 years). The ethnic-racial composition for the sample was as follows: 31.8% Hispanic/Latinx, 31.5% Multiethnic or Multiracial (hereafter Multiethnic), 25.7% White, 7.3% Black or African American, 1.4% Asian American or Pacific Islander (AAPI), 1.4% American Indian or Alaska Native (AI/AN), and 1% Arab, Middle Eastern, or North African (AMENA). The ethnic-racial composition for participants from each school represented overall school level ethnic-racial demographics and was as follows [percentages are rounded]: School 1: 45% Hispanic/Latinx, 27% Multiethnic, 15% White, 7% Black or African American, 2% AAPI, 2% AI/AN, and 1% AMENA; School 2: 48% Hispanic/Latinx, 33% Multiethnic, 3% White, and 16% Black or African American, 0% AAPI, 0% AI/AN, and 0% AMENA; School 3: 11% Hispanic/Latinx, 30% Multiethnic, 53% White, 2% Black or African American, 1% AAPI, 1% AI/AN, and 2% AMENA; and School 4: 28% Hispanic/Latinx, 35% Multiethnic, 26% White, 8% Black or African American, 1% AAPI, 1% AI/AN, and 1% AMENA. Schools 1 (*n* = 222) and 2 (*n* = 58) were middle schools, and schools 3 (*n* = 132) and 4 (*n* = 305) were high schools.

Among Hispanic/Latinx youth in the study sample, 89.5% were of Mexican heritage, 2.5% were Puerto Rican, 1% were Salvadoran, and 6.8% were of another origin. A majority of participants (66.6%) were 3^rd^ generation (i.e., youth and parents born in the U.S.), 17.7% were considered 2.5^th^ generation (i.e., youth and one parent born in the U.S. and the other parent born abroad), 11.8% were 2^nd^ generation (i.e., youth born in the U.S. and both parents born abroad), and 4% were 1^st^ generation (i.e., youth and parents born abroad). Thus, 33.4% of the sample had at least one immigrant or foreign-born parent. Participants reported that their parents were married, never divorced (44.5%), divorced (25.1%), separated (12.2%), widowed (2%), single, never married (8%), or living together but never married (8%).

In terms of subjective appraisal of socioeconomic status, 24.3% of participants reported that they never had to worry about money, 38.2% stated that their family only had to worry about money for fun and extras, 35.1% reported they had just enough to get by, and 2.3% stated that they did not have enough to get by. Thirty-nine percent of participants reported receiving free or reduced-price lunch at school. Participants reported on their parents’ educational levels. Maternal education levels were as follows: 10.4% had less than a high school diploma, 21.3% had a high school diploma or GED, 8.4% had an associate degree, 21.8% had completed some college, 17.1% had a college degree, and 20% had a professional degree (MA, PhD, JD, or MD). Paternal education levels were as follows: 15% had less than a high school diploma, 31.4% had a high school diploma or GED, 7.5% had an associate degree, 18.5% had completed some college, 12% had a college degree, and 15.7% had a professional degree (MA, PhD, JD, or MD).

### Procedure

Participants were 6^th^ grade students from two public middle schools and 9^th^ grade students from two public high schools in a metropolitan city in the Southwestern U.S. Teachers provided all 6^th^ and 9^th^ grade students parental consent letters in English and Spanish to share with their parents/caregivers. Students received $10 for returning their signed parental consent forms to teachers, regardless of their study participation decision. Teachers were provided $50 and two movie tickets for their efforts in reminding students to return consent forms. Participating students with signed parental consent forms provided assent before completing their surveys. Across the four schools, rates of consent ranged from 71% to 81%. These study procedures were approved by the Arizona State University’s institutional review board (Protocol #8845).

Data collection took place in December 2019 and early January 2020. Participants completed self-reported questionnaires in English during their regular school hours over two class periods (approximately 90 minutes total). School staff and research project assistants were available to answer any questions as participants completed the survey.

### Measures

#### School multicultural socialization

Youth rated the extent to which their schools provided opportunities for them to learn about ethno-racial groups and cultures other than their own and about the importance of cultural pluralism using the promotion of cultural competence subscale of the School Climate for Diversity Secondary Scale (Byrd, 2017). Prior work has provided support for the validity and reliability of this subscale among ethno-racially diverse adolescent samples (Byrd, 2017). Youth responded to 5 items (α = 0.92; e.g., “In school you get to do things that help you learn about people of different races and cultures”) based on their experiences in the past six months, on a Likert-type scale from 1 (*not true at all*) to 5 (*completely true*). Raw item scores were recoded to a scale from 0 to 4 to match the scaling of peer and family multicultural socialization measures described below. The average of individual youth scores was calculated, with higher scores indicating greater school multicultural socialization.

#### Peer and family multicultural socialization

Youth were asked how often, in the past six months, their friends/peers and parents/caregivers engaged in efforts to teach them about ethno-racial groups and cultures other than their own and about the importance of equal treatment for people from all ethno-racial backgrounds. Youth responded to a total of six items adapted from the Cultural Socialization/Pluralism subscale of the Parents’ Racial Socialization Scale (Hughes & Johnson, 2001). Prior work has provided support for the validity and reliability of this subscale among ethno-racially diverse adolescent samples (Nelson et al., 2018). Items assessed overt multicultural socialization from peers/friends (3-items; e.g., “Friends/peers talked to you about important people or events in the history of racial/ethnic groups other than your own?” or “Friends/peers have done or said things to show you that all people are equal regardless of race/ethnicity?”) and from parents/caregivers (3-items; e.g., “Parents/Caregivers encouraged you to read books about other racial/ethnic groups?” or “Parents/Caregivers have done or said things to show you that all people are equal regardless of race/ethnicity?”). Response scale ranged from 0 (*never*) to 4 (*very often*). Mean scores were calculated for the friends/peers (α = 0.67) and for the parents/caregivers (α = 0.70) items, with higher scores indicating higher multicultural socialization. The terms peer multicultural socialization and family multicultural socialization will be used hereafter.

#### Academic functioning: Emotional and behavioral academic engagement

Youth reported on two key indicators of academic functioning, namely emotional (4 items; α = 0.90; e.g., “When we work on something in class, I feel interested”) and behavioral academic engagement (6 items; α = 0.91; e.g., “When we work on something in class, I get involved”) using the emotional and behavioral academic engagement subscales from the Engagement versus Disaffection with Learning Scale (Skinner et al., 2009). Prior work has provided support for the validity and reliability of these subscales among ethno-racially diverse adolescent samples (e.g., Martinez-Fuentes et al., 2021). Strong correlations between student and teacher reports and observations of academic engagement further support construct validity (Skinner et al., 2009). The response scale ranged from 0 (*never*) to 4 (*all the time*). Mean scores were calculated, with higher scores indicating higher emotional and behavioral academic engagement.

#### Academic functioning: Academic aspirations and expectations

Youth reported on two additional indicators of academic functioning: academic aspirations and expectations. Questions included how far they would like to go in school (aspirations) and how far they thought they would go in school (expectations). Responses ranged: 1 = *some high school*, 2 = *high school graduate or GED*, 3 = *some college but no degree*, 4 = *graduate from a 2-year college, vocational, or technical school, or join the military*, 5 = *graduate from a four-year college*, 6 = *get an MS/MA*, 7 = *get a professional degree*). Students aspired to attain between a 4-year and a Master’s degree (*M* = 5.24, *SD* = 1.55) and expected to attain between a 2-year and a 4-year degree (*M* = 4.47, *SD* = 1.66).

#### Social position indicators and other covariates

Youth reported on their parents’ nativity or family immigrant status (coded as 1 = *at least one parent born abroad*; 0 = *both parents born in the U.S*.), their gender (1 = *girl*; 0 = *boy* [other cases were coded as missing]), and their ethnicity-race (coded using a series of dummy codes). Students who chose multiple ethnic-racial categories were coded as Multiethnic. School site was treated as a control variable to account for the nested structure of the data and was coded using a series of dummy-codes. Grade was not included as a control because school site was reflective of adolescents’ grade level.

### Data Analysis Plan

Descriptive statistics and bivariate correlations among study variables were examined prior to multivariate analyses. All endogenous variables were normally distributed and did not have outliers. To test study aims, latent profile analyses (LPA) were conducted in *M*plus 8.1 (Muthén, 2004). Across study variables, there were 0% to 8% missing values. To handle the minimal missing data, since imputation is not appropriate for analytical approaches such as LPA, which assumes multiple underlying populations, full-information maximum likelihood (Aims 1 and 2; *n* = 704–682) and listwise deletion (i.e., exploratory Aim 3; *n* = 659) were used. Independent sample t-tests revealed no differences between excluded and kept cases on key study variables.

In Aim 1, this study relied on a person-centered approach (Bergman, 2001) to estimate the latent profiles of U.S. adolescents’ multicultural socialization niches using mean scores of school, peer, and family multicultural socialization as indicators (Suzuki et al., 2021). Specifically, non-diagonal class invariant models, which allow for correlated indicators, were estimated. Solutions with up to 7 profiles were examined and the best-fitting model was selected based on the following criteria: smaller Akaike Information Criterion (AIC; Bozdogan, 1987); Bayesian Information Criterion (BIC; Schwarz, 1978); and sample-size adjusted BIC (aBIC; Sclove, 1987); a significant bootstrapped likelihood ratio test (BLRT; Masyn, 2013) for model K and non-significant BLRT for model K+1, a Bayes Factor (BF; Masyn, 2013) of three at a minimum to indicate at least moderate evidence for Model K compared to Model K+1; a large approximate correct model probability (c*m*P; Masyn, 2013) indicating the probability of a given model being correct out of all fitted models; appraisal of the smallest profile size (Ferguson et al., 2020); and conceptual interpretability of the profiles (Tofighi & Enders, 2008; Weller et al., 2020). Entropy was evaluated as a measure of class categorization but was not used as an indicator in the class enumeration stage (Nylund-Gibson & Choi, 2018).

In Aim 2, the association between the identified latent profile solution and youth academic functioning (i.e., emotional and behavioral academic engagement; academic aspirations and expectations) was examined using the DU3STEP distal outcomes method (Asparouhov & Muthén, 2014; Bakk & Vermunt, 2015). Specifically, Wald tests or mean difference comparisons were estimated between the outcome means (e.g., emotional academic engagement) for each set of pairs (e.g., profile 1 and 2) in the profile solution identified in Aim 1 while accounting for classification error. Significant Wald tests suggest significant mean level differences across the compared profiles.

In exploratory analyses for Aim 3, the associations between social position indicators (i.e., gender, ethnicity/race, and parental nativity) and the identified latent profile solution were examined using the automatic R3STEP three-step approach (Asparouhov & Muthén, 2014; Vermunt, 2010), which estimates multinomial logistic regressions assessing the probability of being in one profile over another. Specifically, the profile solution identified in Aim 1 was regressed on the examined social position variables while accounting for profile classification error and school site. Odds ratio estimates indicate how each of the social position variables are comparatively related to the estimated profiles. Of note, due to the small sample size, cases in which adolescents identified as other gender (1.1%), AAPI (1.4%), AI/AN (1.4%), or AMENA (1%) were omitted from these analyses; therefore, only adolescents who identified as boys, girls, Latinx, Black, Multiethnic, or White were included in these exploratory analyses. For binary indicators, higher social position was coded as 0 (e.g., 1 = girls, 0 = boys). For non-binary indicators, higher social position was identified as the reference group. For example, White adolescents were identified as the reference group given their higher social position status in U.S. society (Loyd & Gaither, 2018) and documented low-levels of related cultural socialization experiences (Abaied & Perry, 2021).

## Results

Table 1 includes descriptive statistics and bivariate correlations for the study variables. School, peer, and family multicultural socialization were positively correlated with one another. Multicultural socialization across settings was positively correlated with emotional and behavioral academic engagement. Family multicultural socialization was positively correlated with academic aspirations and expectations.

**Table 1:** Descriptive Statistics and Bivariate Correlations for Study Variables

		1	2	3	4	5	6	7	8	9	10	11	12	13	14	15	16	*M*	*SD*
*Multicultural Socialization (MCS)*																			
1.	School MCS	--																2.13	1.02
2.	Peer MCS	0.23^***^	--															1.35	0.97
3.	Family MCS	0.25^***^	0.61^***^	--														1.98	1.06
*Academic Functioning*																			
4.	Emotional engagement	0.41^***^	0.17^***^	0.21^***^	--													2.31	0.87
5.	Behavioral engagement	0.33^***^	0.12^**^	0.19^***^	0.67^***^	--												2.87	0.70
6.	Academic aspirations	0.02	0.08^†^	0.10^**^	0.08^*^	0.18^***^	--											5.24	1.55
7.	Academic expectations	0.04	0.10^*^	0.15^***^	0.19^***^	0.31^***^	0.71^***^	--										4.47	1.66
*Social Position Indicators*																		*Frequency*	
8.	Girl ^a^	−0.11^*^	0.04	0.07	−0.17^***^	−0.02	0.25^***^	0.14^**^	--									49.9 %	
9.	Immigrant parent/s ^b^	0.06	0.16^***^	0.13^**^	0.15^***^	0.06	0.08	0.03	0.03	--								33.4 %	
10.	Latinx ^c^	0.07	0.04	0.07	0.07	0.03	−0.09^†^	−0.13^*^	−0.01	0.44^***^	--							31.8 %	
11.	White ^d^	−0.02	−0.03	−0.15^**^	0.02	0.08	0.04	0.14^**^	−0.05	−0.49^***^	--	--						25.7 %	
12.	Black ^e^	0.04	0.02	0.04	−0.03	−0.05	−0.10	−0.14^†^	0.05	0.04	--	--	--					7.3 %	
13.	Multiethnic ^f^	−0.06	−0.04	0.05	−0.07	−0.08	0.04	0.04	0.03	−0.16^*^	--	--	--	--				31.5 %	
14.	AAPI ^g^	0.05	0.02	−0.03	0.17	0.27^†^	0.20	0.05	−0.17	0.46^***^	--	--	--	--	--			1.4 %	
15.	AI/AN ^h^	−0.01	0.01	−0.04	−0.18	−0.19	0.28	−0.06	0.20	−0.31^†^	--	--	--	--	--	--		1.4 %	
16.	AMENA ^i^	−0.12	0.27^†^	0.23^†^	−0.06	0.01	0.27^†^	0.28^†^	0.06	0.62^***^	--	--	--	--	--	--	--	1.0 %	
	*N*	694	657	674	709	710	690	687	699	706	717	717	717	717	717	717	717		
	*% of cases*	97%	92%	94%	99%	99%	96%	96%	97%	98%	100%	100%	100%	100%	100%	100%	100%		

Estimates between continuous variables (1–7) are Pearson’s correlations, between binary variables (8–16) are tetrachoric correlations, and between continuous and binary variables are point-biserial correlations

^a^Girl (1 = *girl*; 0 = *boy*)

^b^Immigrant parent/s (1 = *at least one parent born abroad*; 0 = *both parents born in the U.S*.)

^c^Latinx (1 = *monoracial Hispanic/Latinx*; 0 = *all else*)

^d^White (1 = *monoracial White*; 0 = *all else*)

^e^Black (1 = *monoracial Black or African American*; 0 = *all else*)

^f^Multiethnic (1 = *Multiethnic*; 0 = *all else*)

^g^AAPI (1 = *monoracial Asian American or Pacific Islander*; 0 = *all else*)

^h^AI/AN (1 = *monoracial American Indian or Alaska Native*; 0 = *all else*)

^i^AMENA (1 = *monoracial Arab, Middle Eastern, or North African*; 0 = *all else*)

^†^*p* < 0.10, ^*^p < 0.05, ^**^p < 0.01, ^***^p < 0.001

### Aim 1: Multicultural Socialization Niches

**Fig. 1:**
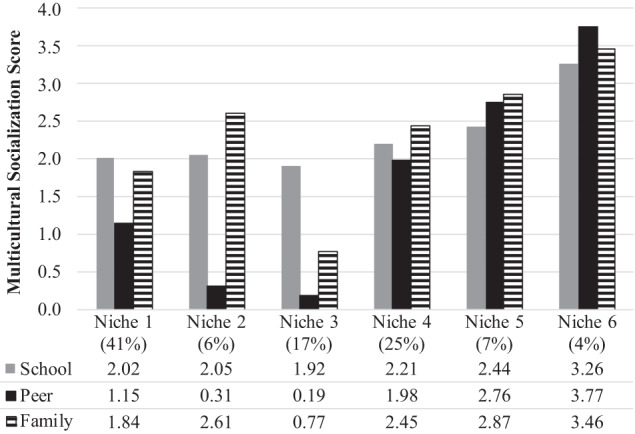
Aim 1: Means and Proportions for the 6-Profile LPA Solution of Multicultural Socialization Niches (*n* = 704). *Note*. Niche 1: Cross-setting dissimilar peer contrast socialization niche (*n* = 286); Niche 2: Cross-setting dissimilar greater peer contrast socialization niche (*n* = 41); Niche 3: Cross-setting dissimilar school contrast socialization niche (*n* = 119); Niche 4: Cross-setting similar lower socialization niche (*n* = 177); Niche 5: Cross-setting similar moderate socialization niche (*n* = 52); and Niche 6: Cross-setting similar higher socialization niche (*n* = 29). Average multicultural socialization scores are depicted under niche labels. Final counts for the latent profiles are based on classified profile membership

The six-profile LPA solution was selected because it was the best-fitting model with good interpretability based on theory (Bronfenbrenner & Morris, 2006; Super & Harkness, 2002; Table 2). Specifically, the six-profile solution had lower AIC, BIC, and adjusted BIC values and a higher c*m*P value than all the other solutions. Further, compared to the seven-profile solution, the six-profile solution had a statistically significant BLRT value and a BF value larger than 10, providing further evidence for the six-profile LPA solution as the best-fitting model. Supporting Hypothesis 1, the six identified profiles or niches were characterized by different types of degree and cross-setting consistency of multicultural socialization experiences. In this model (see Fig. 1), three niches (Niches 4, 5, 6) demonstrated relatively similar patterns across school-peer-family (i.e., mean level differences across settings within each niche were not greater than 0.51), indicating cross-setting similarity in multicultural socialization experiences within each niche but these three niches ranged in the degree or level to which youth were afforded socialization opportunities (i.e., lower to higher). Roughly 25% of adolescents were in the *cross-setting similar lower socialization* niche (Niche 4; *n* = 177), which was characterized by lower levels (i.e., mean levels between 1.98 and 2.45) of school-peer-family multicultural socialization. Approximately 7% of adolescents were in the *cross-setting similar moderate socialization* niche (Niche 5; *n* = 52), which was defined by moderate levels (i.e., mean levels between 2.44 and 2.87) of school-peer-family multicultural socialization. Only 4% of adolescents were in the *cross-setting similar higher socialization* niche (Niche 6; *n* = 29), which was characterized by the highest levels (i.e., mean levels between 3.26 and 3.77) of school-peer-family multicultural socialization. This profile represents a meaningful group that also emerged in the other profile solutions (e.g., Appendix A).

**Table 2:** Aim 1: Model Fit Criteria for Latent Profile Analysis of Multicultural Socialization Niches (*n* = 704)

Index	1 profile	2 profiles	3 profiles	4 profiles	5 profiles	6 profiles	7 profiles
Loglikelihood	−2730.14	−2709.20	−2686.41	−2668.94	−2653.85	−**2636.66**	−2641.43
Parameters	9	13	17	21	25	**29**	33
AIC	5478.28	5444.39	5406.82	5379.89	5357.70	**5331.32**	5348.87
BIC	5519.29	5503.63	5484.29	5475.58	5471.62	**5463.47**	5499.24
aBIC	5490.71	5462.35	5430.31	5408.90	5392.24	**5371.39**	5394.46
Entropy	1.00	0.77	0.63	0.64	0.75	**0.79**	0.70
Δ AIC	--	−33.89	−37.57	−26.94	−22.19	−**26.38**	17.55
Δ BIC	--	−15.66	−19.34	−8.71	−3.96	−**8.15**	35.77
Δ aBIC	--	−28.36	−32.04	−21.41	−16.66	−**20.85**	23.07
BLRT	--	< 0.001	< 0.001	< 0.001	< 0.001	**< 0.001**	0.07
BF	3.97E-04	6.32E-05	1.28E-02	1.38E-01	1.70E-02	**5.86E+07**	--
c*m*P	7.41E-13	1.87E-09	2.95E-05	2.30E-03	1.67E-02	**9.81E-01**	1.67E-08
Smallest profile (% of sample) ^a^		57 (8%)	87 (12%)	40 (6%)	31 (4%)	**29 (4%)**	14 (2%)

*AIC* Akaike Information Criterion, *BIC* Bayesian Information Criterion, *aBIC* Adjusted BIC, *BLRT* Bootstrapped Likelihood Ratio Test, *BF* Bayes Factor, *cmP* Approximate Correct Model Probability. Lower AIC, BIC, and aBIC values and a BF value that is large and at least 3 represent better fit. A larger c*m*P value represents a larger probability of a given model is correct out of all fitted models. A significant BLRT for model K and non-significant BLRT for model K+1 indicates that the model with K profiles is a better fitting model. *Boldface* indicates the solution that was selected as the best fitting model

^a^Counts and proportions for the smallest latent profile are based on the estimated model’s probabilistic likelihood of profile membership

The remaining three niches (Niches 1, 2, 3) demonstrated relatively dissimilar patterns across school-peer-family (i.e., mean level differences across at least two contrasting settings within each profile were greater than 0.69) indicating cross-setting dissimilarity, which ranged in the type of contrast in multicultural socialization experiences across each of the settings (e.g., greater mean level differences were found in the school setting vs. the other settings) within niches and the degree or level to which youth were afforded socialization opportunities (i.e., lower to moderate). A large proportion (41%) of adolescents were in the *cross-setting dissimilar peer contrast socialization* niche (Niche 1; *n* = 286), which was characterized by greater dissimilarities in multicultural socialization experiences between peer (*M* = 1.15) and the other settings (*M*_*School*_ = 2.02; *M*_*Family*_ = 1.84) ranging between very low to low levels of multicultural socialization across settings. Roughly 6% adolescents were in the *cross-setting dissimilar greater peer contrast socialization* niche (Niche 2; *n* = 41), which was characterized by the highest dissimilarity in multicultural socialization experiences between peer (*M* = 0.31) and the other settings (*M*_*School*_ = 2.05, *M*_*Family*_ = 2.61) ranging from very low to moderate levels of multicultural socialization across settings. Approximately 17% of adolescents were in the *cross-setting dissimilar school contrast socialization* niche (Niche 3; *n* = 119), which was characterized by greater dissimilarities in multicultural socialization experiences between school (*M* = 1.92) and the other settings (*M*_*Peer*_ = 0.19; *M*_*Family*_ = 0.77) and the lowest levels of multicultural socialization across each setting. In sensitivity analyses (Appendix A), findings for the five-profile solution were examined. Overall, similar profiles or niches were estimated, but the cross-setting similar moderate multicultural socialization niche did not emerge in the five-profile solution.

### Aim 2: Associations Between Multicultural Socialization Niches and Academic Functioning

The second research aim examined how the multicultural socialization niches identified in Aim 1 were related to markers of academic functioning (Table 3). Supporting Hypothesis 2a, mean comparisons for the six niches revealed that emotional academic engagement was significantly higher for adolescents negotiating the *cross-setting similar higher socialization* niche (Niche 6) compared to adolescents negotiating the other five niches (Niches 1 through 5). Similarly, behavioral academic engagement was also greater for adolescents in this niche compared to adolescents in all other niches, except those in the *cross-setting dissimilar greater peer contrast* niche (Niche 2). Further, emotional academic engagement was significantly higher for adolescents negotiating the *cross-setting similar moderate socialization* niche (Niche 5) compared to adolescents negotiating the *cross-setting dissimilar peer contrast* (Niche 1) and *school contrast* (Niche 3) multicultural socialization niches.

**Table 3:** Aim 2: Associations Between the 6-Profile LPA Solution of Multicultural Socialization Niches and Academic Functioning

	Emotional Academic Engagement (*n* = 701)		Behavioral Academic Engagement (*n* = 702)		Academic Aspirations (*n* = 683)		Academic Expectations (*n* = 682)	
Academic Functioning Means by Niche	*M*	*SE*	*M*	*SE*	*M*	*SE*	*M*	*SE*
1. Cross-setting dissimilar peer contrast socialization niche	2.190	0.062	2.780	0.053	5.216	0.127	4.446	0.129
2. Cross-setting dissimilar greater peer contrast socialization niche	2.493	0.208	3.131	0.132	4.995	0.370	4.763	0.328
3. Cross-setting dissimilar school contrast socialization niche	2.133	0.095	2.788	0.076	5.029	0.178	4.147	0.166
4. Cross-setting similar lower socialization niche	2.359	0.076	2.853	0.062	5.423	0.147	4.673	0.153
5. Cross-setting similar moderate socialization niche	2.504	0.130	2.996	0.121	5.427	0.230	4.414	0.278
6. Cross-setting similar higher socialization niche	3.111	0.143	3.416	0.076	5.189	0.219	4.988	0.304
**Wald Tests Comparisons Across Niches**	χ ^2^ (1) =	*d*	χ ^2^ (1) =	*d*	χ ^2^ (1) =	*d*	χ ^2^ (1) =	*d*
*6. Cross-setting similar higher socialization niche vs*.:								
1. Cross-setting dissimilar peer contrast socialization niche	34.438^***^	**0.898**	46.300^***^	**0.736**	0.011	−0.013	2.662	0.254
2. Cross-setting dissimilar greater peer contrast socialization niche	5.994^*^	**0.545**	3.476 ^†^	0.414	0.205	0.098	0.254	0.117
3. Cross-setting dissimilar school contrast socialization niche	32.281^***^	**0.989**	34.049^***^	**0.819**	0.322	0.088	5.905^*^	**0.474**
4. Cross-setting similar lower socialization niche	21.665^***^	**0.767**	32.643^***^	**0.725**	0.800	−0.125	0.871	0.162
5. Cross-setting similar moderate socialization niche	8.901^**^	**0.692**	8.373^**^	**0.570**	0.520	−0.156	1.682	0.304
*5. Cross-setting similar moderate socialization niche vs.:*								
1. Cross-setting dissimilar peer contrast socialization niche	4.716^*^	**0.304**	2.676	0.242	0.656	0.104	0.011	−0.015
2. Cross-setting dissimilar greater peer contrast socialization niche	0.002	0.012	0.567	−0.157	0.982	0.245	0.658	−0.170
3. Cross-setting dissimilar school contrast socialization niche	5.299^*^	**0.368**	2.126	0.248	1.847	0.231	0.679	0.142
4. Cross-setting similar lower socialization niche	0.824	0.146	0.981	0.175	0.000	0.002	0.588	−0.129
*4. Cross-setting similar lower socialization niche vs.:*								
1. Cross-setting dissimilar peer contrast socialization niche	2.520	0.163	0.646	0.080	0.891	0.093	1.071	0.107
2. Cross-setting dissimilar greater peer contrast socialization niche	0.368	−0.121	3.632 ^†^	−0.339	1.175	0.181	0.062	−0.045
3. Cross-setting dissimilar school contrast socialization niche	3.522 ^†^	0.221	0.451	0.075	3.063 ^†^	0.179	5.554^*^	**0.273**
*3. Cross-setting dissimilar school contrast socialization niche vs.:*								
1. Cross-setting dissimilar peer contrast socialization niche	0.221	−0.055	0.006	0.009	0.623	−0.066	1.784	−0.144
2. Cross-setting dissimilar greater peer contrast socialization niche	2.065	−0.318	4.235^*^	**−0.412**	0.006	0.009	2.394	−0.326
*2. Cross-setting dissimilar greater peer contrast socialization niche vs.:*								
1. Cross-setting dissimilar peer contrast socialization niche	1.856	0.275	5.708^*^	**0.394**	0.300	−0.109	0.760	0.146

*M* Mean, *SE* Standard Error, *VS.* Versus. Boldface represents significant Wald tests (*p* < 0.05) indicating mean level differences across compared niches

^†^*p* < 0.10, ^*^p < 0.05, ^**^p < 0.01, ^***^p < 0.001

Partially supporting Hypothesis 2b, mean comparisons revealed that adolescents negotiating the *cross-setting dissimilar school contrast socialization* niche (Niche 3), which was also characterized by the lowest levels of multicultural socialization in each of the settings, demonstrated lower emotional academic engagement compared to adolescents negotiating the *cross-setting similar higher* (Niche 6) and *moderate* (Niche 5) socialization niches. These adolescents also showed lower behavioral academic engagement compared to adolescents negotiating the *cross-setting similar higher* (Niche 6) and *dissimilar greater peer contrast* (Niche 2) socialization niches; lower academic expectations compared to adolescents in the *cross-setting similar higher* (Niche 6) and *lower* (Niche 4) socialization niches. Similarly, behavioral academic engagement was also lower for adolescents negotiating the *cross-setting dissimilar peer contrast socialization* niche (Niche 1), which was characterized by the second lowest levels of multicultural socialization in each of the settings, compared to adolescents in the *cross-setting dissimilar greater peer contrast* niche (Niche 2). No significant differences in academic aspirations were found across niches.

### Aim 3 Exploratory Analyses: Social Position Predictors of Multicultural Socialization Niches

Exploratory analyses examined the associations between salient social position indicators (i.e., gender, ethnicity/race, and parental nativity) and the identified multicultural socialization niches (Table 4; see Appendix B for a description of the niches by social position indicators) while accounting for school site. Findings from multinomial logistic regression analyses indicated that social position was a significant predictor of profile membership above and beyond any school site effects. In terms of gender, girls had lower odds or were less likely than boys to be classified in the *cross-setting dissimilar peer contrast* (Niche 1) and *school contrast* (Niche 3) socialization niches and in the *cross-setting similar lower socialization* niche (Niche 4) compared to the *cross-setting similar moderate socialization* niche (Niche 5). Similarly, girls had higher odds than boys to be in the *cross-setting similar moderate* niche (Niche 5) compared to the *cross-setting similar higher socialization* niche (Niche 6). Taken together, these findings suggest that girls were more likely to negotiate the *cross-setting similar moderate socialization* niche (Niche 5) compared to most niches (i.e., Niches 1, 3, 4, 6).

**Table 4:** Exploratory Aim 3: Multinomial Logistic Regression Analyses: Associations Between Social Position and the 6-Profile LPA Solution of Multicultural Socialization Niches (*n* = 659)

	Niches 1 vs. 2			Niches 1 vs. 3			Niches 1 vs. 4		
	Coef.	SE	OR	Coef.	SE	OR	Coef.	SE	OR
Girl^a^	−0.062	0.484	0.940	0.262	0.287	1.300	0.082	0.272	1.085
Immigrant parent/s^b^	−0.348	0.518	0.706	−0.104	0.355	0.901	−0.397	0.318	0.672
Latinx	−0.807	0.707	0.446	0.542	0.414	1.719	0.001	0.395	1.001
Black	−1.324	0.929	0.266	0.267	0.707	1.306	−0.486	0.591	0.615
Multiethnic	0.039	0.779	1.040	0.238	0.359	1.269	0.038	0.366	1.039
White^c^	--	--	--	--	--	--	--	--	--
School 1	−0.360	0.514	0.698	−0.505	0.344	0.604	−0.055	0.333	0.946
School 2	1.196	2.202	3.307	−1.084^†^	0.559	0.338	−0.241	0.565	0.786
School 3	−0.118	0.706	0.889	−0.075	0.405	0.928	−0.077	0.388	0.926
School 4^d^	--	--	--	--	--	--	--	--	--
	Niches 1 vs. 5			Niches 1 vs. 6			Niches 2 vs. 3		
	Coef.	SE	OR	Coef.	SE	OR	Coef.	SE	OR
Girl^a^	−1.092^**^	**0.407**	**0.336**	1.099^**†**^	0.564	3.001	0.324	0.544	1.383
Immigrant parent/s^b^	−1.145^**^	**0.391**	**0.318**	−1.137^*^	**0.564**	**0.321**	0.244	0.587	1.276
Latinx	−1.016	0.662	0.362	1.500^*^	**0.700**	**4.482**	1.349^**†**^	0.772	3.854
Black	−0.353	1.096	0.703	0.053	0.816	1.054	1.591	1.079	4.909
Multiethnic	−1.202^**†**^	0.627	0.301	2.972^*^	**1.409**	**19.531**	0.199	0.834	1.220
White ^c^	--	--	--	--	--	--	--	--	--
School 1	−0.078	0.433	0.925	−0.550	0.664	0.577	−0.145	0.574	0.865
School 2	−0.192	0.680	0.825	−1.943^*^	**0.823**	**0.143**	−2.281	2.283	0.102
School 3	−0.466	0.519	0.628	−0.705	0.678	0.494	0.043	0.771	1.044
School 4^d^	--	--	--	--	--	--	--	--	--
	Niches 2 vs. 4			Niches 2 vs. 5			Niches 2 vs. 6		
	Coef.	SE	OR	Coef.	SE	OR	Coef.	SE	OR
Girl^a^	0.144	0.481	1.155	−1.030^**†**^	0.576	0.357	1.161^**†**^	0.699	3.193
Immigrant parent/s^b^	−0.049	0.507	0.952	−0.797	0.569	0.451	−0.789	0.702	0.454
Latinx	0.808	0.706	2.243	−0.208	0.895	0.812	2.308^*^	**0.927**	**10.054**
Black	0.838	0.892	2.312	0.971	1.297	2.641	1.377	1.081	3.963
Multiethnic	−0.002	0.783	0.998	−1.242	0.942	0.289	2.933^**†**^	1.580	18.784
White^c^	--	--	--	--	--	--	--	--	--
School 1	0.305	0.509	1.357	0.282	0.591	1.326	−0.189	0.778	0.828
School 2	−1.438	2.185	0.237	−1.389	2.209	0.249	−3.140	2.272	0.043
School 3	0.041	0.707	1.042	−0.348	0.793	0.706	−0.587	0.913	0.556
School 4 ^d^	--	--	--	--	--	--	--	--	--
	Niches 3 vs. 4			Niches 3 vs. 5			Niches 3 vs. 6		
	Coef.	SE	OR	Coef.	SE	OR	Coef.	SE	OR
Girl^a^	−0.180	0.290	0.835	−1.354^**^	**0.434**	**0.258**	0.837	0.577	2.309
Immigrant parent/s^b^	−0.293	0.340	0.746	−1.040^*^	**0.431**	**0.353**	−1.033^**†**^	0.579	0.356
Latinx	−0.541	0.417	0.582	−1.557^*^	**0.699**	**0.211**	0.959	0.718	2.609
Black	−0.753	0.641	0.471	−0.620	1.152	0.538	−0.215	0.858	0.807
Multiethnic	−0.201	0.372	0.818	−1.441^*^	**0.653**	**0.237**	2.734^**†**^	1.410	15.394
White^c^	--	--	--	--	--	--	--	--	--
School 1	0.450	0.348	1.568	0.427	0.471	1.533	−0.044	0.679	0.957
School 2	0.843	0.516	2.323	0.892	0.692	2.440	−0.859	0.810	0.424
School 3	−0.002	0.415	0.998	−0.391	0.569	0.676	−0.630	0.704	0.533
School 4^d^	--	--	--	--	--	--	--	--	--
	Niches 4 vs. 5			Niches 4 vs. 6			Niches 5 vs. 6		
	Coef.	SE	OR	Coef.	SE	OR	Coef.	SE	OR
Girl^a^	−1.174^**^	**0.440**	**0.309**	1.017^**†**^	0.567	2.765	2.190^**^	**0.682**	**8.935**
Immigrant parent/s^b^	−0.747^**†**^	0.421	0.474	−0.740	0.561	0.477	0.008	0.656	1.008
Latinx	−1.017	0.707	0.362	1.500^*^	**0.709**	**4.482**	2.516^**^	**0.929**	**12.379**
Black	0.133	1.123	1.142	0.538	0.789	1.713	0.406	1.259	1.501
Multiethnic	−1.240^**†**^	0.675	0.289	2.935^*^	**1.409**	**18.822**	4.175^**^	**1.555**	**65.040**
White^c^	--	--	--	--	--	--	--	--	--
School 1	−0.023	0.474	0.977	−0.495	0.670	0.610	−0.472	0.773	0.624
School 2	0.049	0.737	1.050	−1.702^*^	**0.805**	**0.182**	−1.751^**†**^	0.963	0.174
School 3	−0.390	0.575	0.677	−0.628	0.692	0.534	−0.238	0.827	0.788
School 4^d^	--	--	--	--	--	--	--	--	--

*VS.* Versus, *Coef.* Coefficient, *SE* standard error, *OR* odds ratio. **Boldface** represents significant estimates (*p* < 0.05) indicating that a given social position indicator was a significant predictor of profile membership across compared niches, specifically estimates reflect the effects of the predictors on the likelihood of membership into the first versus second listed niche. Niche 1: Cross-setting dissimilar peer contrast socialization; Niche 2: Cross-setting dissimilar greater peer contrast socialization; Niche 3: Cross-setting dissimilar school contrast socialization; Niche 4: Cross-setting similar lower socialization; Niche 5: Cross-setting similar moderate socialization; and Niche 6: Cross-setting similar higher socialization

^a^Girl (1 = *girl*; 0 = *boy*)

^b^Immigrant parent/s (1 = *at least one parent born abroad*; 0 = *both parents born in the U.S*.)

^c^White was coded as the reference group across model comparisons examining the role of ethnicity/race

^d^School 4 (largest sample size) was coded as the reference group across model comparisons and treated as a control variable to account for the nested structure of data

^†^*p* < 0.10, ^*^p < 0.05, ^**^p < 0.01

Turning to ethnicity/race, compared to White adolescents, Latinx and Multiethnic adolescents had higher odds to be in the *cross-setting dissimilar peer contrast socialization* niche (Niche 1) and in the *cross-setting similar lower* (Niche 4) and *moderate* (Niche 5) socialization niches compared to the *cross-setting similar higher socialization* niche (Niche 6). They were also less likely to be in the *cross-setting dissimilar school contrast socialization* niche (Niche 3) compared to the *cross-setting similar moderate socialization* niche (Niche 5). Compared to White youth, Latinx adolescents were more likely to be in the *cross-setting dissimilar greater peer contrast* niche (Niche 2) than the *cross-setting similar higher socialization* niche (Niche 6). There were no differences in the likelihood of profile membership between White and Black adolescents. Together, these findings indicate that ethnicity/race informs the multicultural socialization niches that adolescents negotiate on regular basis, particularly for Latinx and Multiethnic youth when compared to White youth.

In terms of parental nativity, adolescents with at least one immigrant parent (i.e., foreign-born) had lower odds or were less likely than adolescents with U.S.-born parents to be classified in the *cross-setting dissimilar peer contrast* (Niche 1) and *school contrast* (Niche 3) socialization niches compared to the *cross-setting similar moderate socialization* niche (Niche 5) and less likely to be in the *cross-setting dissimilar peer contrast socialization* niche (Niche 1) compared to the *cross-setting similar higher socialization* niche (Niche 6). Taken together, these findings suggest that adolescents with immigrant parents were less likely to negotiate cross-setting dissimilar niches (i.e., Niches 1, 3) compared to cross-setting similar niches (i.e., Niches 5, 6).

School site was treated as a control variable. Overall, there were no differences in the likelihood of profile membership between adolescents in School 4 (largest sample size) and those in the other three schools. However, compared to School 4, adolescents attending School 2 (smallest sample size) had lower odds or were less likely to be negotiating the *cross-setting dissimilar peer contrast* (Niche 1) and *cross-setting similar lower* (Niche 4) socialization niches compared to the *cross-setting similar higher socialization* niche (Niche 6).

## Discussion

Cultural diversity characterizes many parts of the world (Pew Research Center, 2019, April 22) and has important implications for the development and adjustment of youth from all ethno-racial groups (Berry et al., 2022), particularly for their academic adjustment as youth increasingly attend ethno-racially diverse schools (Nishina et al., 2019). Nevertheless, there is scarce research on how socialization processes equip youth to respond to increasing multicultural demands and the degree to which these socialization experiences inform youth academic functioning. This study addressed this gap by examining multicultural socialization niches across key proximal settings (i.e., schools, peers, and families) and their links with youth academic functioning. Consistent with ecological models highlighting unique contexts of development (e.g., Bronfenbrenner & Morris, 2006), this study identified a range of multicultural socialization niches that adolescents regularly negotiate: *cross-setting similar higher, moderate*, *and lower socialization niches* and *cross-setting dissimilar peer contrast*, *greater peer contrast*, *and school contrast socialization niches*. Most adolescents were negotiating niches in which they were afforded lower and/or dissimilar multicultural socialization opportunities (Aim 1). Further, findings suggest that contextual diversity matters as both the degree and consistency characterizing youth multicultural socialization niches had implications for their academic functioning, particularly more cohesive niches seemed the most beneficial (Aim 2). In line with theoretical notions underscoring the role that social stratification mechanisms play on youth development (e.g., García Coll et al., 1996), results from exploratory analyses suggest that indicators of youth social position may also shape the multicultural socialization niches that adolescents navigate (Aim 3).

### School–Peer–Family Multicultural Socialization Niches (Aim 1)

Building on ecological models that consider the unique, intersecting nature of the developmental contexts youth regularly negotiate (e.g., Bronfenbrenner & Morris, 2006), this study examined variability in adolescent multicultural socialization niches relative to the degree and consistency of multicultural socialization experiences afforded to them across school, peer, and family settings. This approach recognizes the importance of these settings across adolescence (Eccles & Roeser, 2011) and their substantial interactions (Bronfenbrenner & Morris, 2006). Further, it acknowledges that socialization niches emerge from adaptive cultural models reflecting societal and individual values (White et al., 2018) and are influenced by the beliefs and practices that characterize a given setting (Super & Harkness, 2002). Six distinct socialization niches were identified using a person-centered approach (Bergman, 2001) and supporting Hypothesis 1. Consistent with prior work focused on related types of cultural socialization experiences (i.e., heritage and national cultural socialization; combination of cultural socialization experiences) across school and family settings (Byrd & Ahn, 2020) and across peer and family settings (Wang & Benner, 2016), findings from the current study highlight that U.S. adolescents from multiple ethno-racial backgrounds are negotiating a diverse range of multicultural socialization niches that vary in the *degree* and *consistency/similarity* in socialization experiences across school, peer, and family settings.

Three niches demonstrated cross-setting similarity and ranged in the degree or level youth were afforded socialization opportunities. Greater levels of school-peer-family multicultural socialization characterized the *cross-setting similar higher socialization* niche compared to the *cross-setting similar moderate* and *lower socialization* niches, characterized by moderate and lower levels of multicultural socialization, respectively. There was a lot of variability in the number of adolescents negotiating each of these niches. Specifically, the *cross-setting similar lower socialization* niche represented a quarter of adolescents. In contrast, the *cross-setting similar moderate* and *higher socialization* niches included only seven and four percent of adolescents, respectively.

Across these cross-setting similar niches, adolescents likely encounter comparable cross-setting messages and opportunities to learn about and treat with respect members of multiple cultures. Further, adolescents may be able to draw additional benefits from socialization experiences taking place in cohesive niches, particularly when given ample socialization opportunities, because the mutually reinforcing repetition of similar influences occurring across school-peer-family settings can better support youth in internalizing these messages and developing multicultural competencies (Super & Harkness, 2002). Albeit small, the *cross-setting similar higher* and *moderate socialization* niches represent important niches. Indeed, prior work has documented comparable niches and proportions. For instance, work on related types of cultural socialization (i.e., heritage and national cultural socialization; combination of cultural socialization experiences) has documented the significance of frequent, cross-setting similar cultural socialization experiences across school and family settings (Byrd & Ahn, 2020) and across peer and family settings (Wang & Benner, 2016). Consistent with the current study, this work also found the cross-setting similar higher niches to represent small proportions of their samples (Byrd & Ahn, 2020), perhaps because historical assimilationist practices in U.S. schooling and other settings make it unlikely to observe high and similar levels of multicultural socialization across settings (Urrieta & Machado-Casas, 2013).

Three niches demonstrated cross-setting dissimilarity which ranged in the type of cross-setting contrast and the degree to which youth were afforded socialization opportunities. The *cross-setting dissimilar school contrast socialization* niche was characterized by greater dissimilarities between the multicultural socialization experiences afforded to youth in the school setting compared to the peer and family settings and demonstrated the lowest levels of cross-setting multicultural socialization of all niches. The other two niches, the *cross-setting dissimilar peer contrast* and *greater peer contrast socialization* niches were characterized by larger dissimilarities between the multicultural socialization experiences provided to youth in the peer setting compared to the school and family settings. In the former, however, the contrast was lower, and cross-setting multicultural socialization experiences ranged from very low to low. In the latter, the contrast was higher and cross-setting multicultural socialization experiences ranged from very low to moderate levels. The *cross-setting dissimilar peer contrast* niche was the largest niche representing 41 percent of adolescents whereas the *cross-setting dissimilar greater peer contrast* and *school contrast* niches included a six and a 17 percent of adolescents, respectively.

In these cross-setting dissimilar niches, adolescents are likely exposed to competing messages across educators, peers, and caregivers regarding the importance of cultural pluralism and equal treatment of members of all ethno-racial groups. Conflicting messages may diminish adolescents’ ability to develop multicultural competencies (Ward & Szabó, 2023). Further, this lack of cohesiveness across salient developmental settings may prove affectively, behaviorally, and cognitively taxing (Safa et al., 2019); thus, may reduce the benefits adolescents can draw from these socialization experiences. Indeed, prior work on related types of cultural socialization (i.e., heritage and national cultural socialization; combination of cultural socialization experiences) has documented developmental costs of dissimilar cultural socialization experiences across school and family settings (Byrd & Ahn, 2020) and across peer and family settings (Wang & Benner, 2016) to adolescent psychosocial adjustment.

It is not surprising that most adolescents in the current sample are negotiating cross-setting dissimilar or cross-setting similar lower multicultural socialization niches. Indeed, creating a harmonious, culturally plural society where people from all ethno-racial groups are valued and treated equally is a desirable (Deaux & Verkuyten, 2014) but complex goal (Berry et al., 2022). Further, many current U.S. state policies (e.g., HB 3979 in Texas, SB 1070 in Arizona) are inconsistent with multiculturalism values and the effects of these policies trickle down to the proximal settings youth navigate, including schools and families (Santos et al., 2018). Finally, socialization agents such as teachers (Chahar Mahali & Sevigny, 2022) and parents (Anderson & Stevenson, 2019) often report not being equipped to provide multicultural socialization opportunities to youth in rapidly changing settings within diverse communities. Thus, the proportion of adolescents negotiating the identified niches may exemplify constraints faced by schools, peers, and families in aligning values and goals related to multiculturalism. These constraints are likely imposed by historical systems and derivatives of social stratification including racism, discrimination, and segregation (García Coll et al., 1996).

### Multicultural Socialization Niches and Youth Academic Functioning (Aim 2)

In line with ecological models underscoring the intersecting influence of proximal contexts on youth adjustment (e.g., Bronfenbrenner & Morris, 2006), this study examined the role of youth multicultural socialization niches on their academic functioning. Supporting Hypothesis 2a, youth negotiating niches characterized by cross-setting similarity with relatively higher levels or degree of multicultural socialization demonstrated better academic functioning than youth in other niches. Specifically, adolescents negotiating the *cross-setting similar higher socialization* niche had greater emotional academic engagement than adolescents in the other five niches. These adolescents also demonstrated higher behavioral academic engagement than adolescents in all other niches except those in the *cross-setting dissimilar greater peer contrast* niche. In addition, adolescents negotiating the *cross-setting similar moderate socialization* niche also demonstrated higher emotional academic engagement than adolescents negotiating the *cross-setting dissimilar peer contrast* and *school contrast socialization* niches. These findings highlight the importance of cross-setting similarity and moderate-to-higher levels of multicultural socialization for adolescent academic engagement. These results are consistent with theoretical notions underscoring the adjustment-related benefits of cohesiveness (Bronfenbrenner & Morris, 2006) and mutually reinforcing repetition (Super & Harkness, 2002) across adolescent proximal contexts of development.

It is likely that adolescents negotiating cohesive niches with at least moderate levels of multicultural socialization are afforded relatively consistent and frequent opportunities across schools, peers, and families. These opportunities can help youth to understand the benefits and challenges of cultural pluralism (Berry et al., 2022), to negotiate everyday interactions with people from culturally diverse backgrounds (Neblett et al., 2012), to develop a sense of belonging in culturally plural settings (Nishina et al., 2019), and to gain other multicultural competencies needed to navigate culturally diverse academic settings and demands (García Coll & Szalacha, 2004). Thus, the socialization opportunities afforded to youth in these moderately and highly multicultural socialization niches can foster the development of self-concept and skills to successfully navigate increasingly diverse educational settings and demands (Saleem & Byrd, 2021), which can bolster their behavioral and academic engagement in schools.

The current study extends prior work documenting the importance of adolescent cross-setting similar higher cultural socialization (e.g., heritage, national, or a combination) niches for their academic adjustment (peer and family settings; Wang & Benner, 2016) and academic engagement and aspirations (Byrd & Ahn, 2020) by focusing on the interactive influence of three key proximal contexts during adolescence (i.e., schools, peers, and families) on a less studied but increasingly salient socialization process, namely *multicultural socialization*. Nevertheless, findings should be interpreted cautiously as they present limited evidence of the benefits of cross-setting similar higher and moderate multicultural socialization niches in a small proportion of the sample. In addition, the fact that there were no differences in emotional academic engagement between youth in the *cross-setting similar higher* and *cross-setting dissimilar greater peer contrast* niches, the latter was the cross-setting dissimilar niche with the highest levels of family multicultural socialization, may suggest that multicultural socialization opportunities taking place in the family setting are particularly promotive of youth academic engagement. More work is needed to understand the benefits of cross-setting consistency and the optimal degree of multicultural socialization experiences within specific settings for youth academic functioning.

Hypothesis 2b was partially supported as adolescents negotiating the *cross-setting dissimilar school contrast socialization* niche, which was the niche characterized by cross-setting dissimilarity and lowest levels of multicultural socialization, demonstrated lower academic functioning than adolescents in some of the other niches. Importantly, most differences emerged between this niche and the cross-setting similar socialization niches. Specifically, youth in the *cross-setting dissimilar school contrast socialization* niche demonstrated lower emotional (vs. *cross-setting similar higher* and *moderate socialization* niches) and behavioral (vs. *cross-setting similar higher socialization* niche) academic engagement and lower academic expectations (vs. *cross-setting similar higher* and *lower socialization* niches). Comparisons with the other cross-setting dissimilar niches revealed that adolescents in this niche showed lower behavioral academic engagement than adolescents negotiating the *cross-setting dissimilar greater peer contrast socialization* niche. Relatedly, adolescents negotiating the *cross-setting dissimilar peer contrast socialization* niche, characterized by the second lowest levels of cross-setting socialization, also demonstrated lower behavioral academic engagement than adolescents in the *cross-setting dissimilar greater peer contrast* niche. Consistent with theoretical notions (Bronfenbrenner & Morris, 2006), these findings exemplify the cost of lack of cohesiveness across youth developmental contexts and of fragmented multicultural socialization opportunities for adolescent academic engagement and expectations. Of note, prior work on related types of cultural socialization (i.e., heritage and national cultural socialization; combination of cultural socialization experiences) did not find any differences in academic functioning between adolescents in the cross-setting dissimilar niches and those in the lower-level cross-setting similar niches (e.g., Byrd & Ahn, 2020). However, findings from the current study suggest that inconsistency combined with lower levels of multicultural socialization is most detrimental to youth academic engagement and expectations. Future work should continue to examine the developmental implications of contrasting socialization experiences across settings involving different degrees of socialization efforts.

It is likely that adolescents negotiating dissimilar niches with lower levels of multicultural socialization are afforded scarce and/or conflicting opportunities across schools, peers, and families to learn about the importance of cultural pluralism and equal treatment for members of all ethno-racial groups. Infrequent and inconsistent opportunities may result in limited opportunities for youth to develop behavioral, cognitive, and social skills to navigate ethno-racially diverse settings and a lack of efficacy in responding to multicultural demands (Wang & Benner, 2016). Further, these adolescents may engage in substantial efforts to reconcile inconsistent messages and to alter their behaviors to meet the demands of specific settings which could prove behaviorally, cognitively, and socially taxing (Safa et al., 2019), and this, in turn, may reduce their academic functioning (Safa et al., 2022).

Notably, contextual diversity of the multicultural socialization niches captured in this study informed youth emotional and behavioral academic engagement. However, the range of diversity in consistency and degree of cross-setting multicultural socialization opportunities minimally informed their academic expectations and did not inform their academic aspirations at all. It is likely that youth social position including socioeconomic status and parental education constrained the benefits of multicultural socialization opportunities for youth academic aspirations and expectations. Indeed, prior work has documented that indicators of social position such as parent educational attainment have important implications for youth’s educational aspirations and expectations because they provide youth with funds of knowledge and opportunities to aspire and pursue their academic goals (e.g., Lui et al., 2014). Alternatively, the development of self-concept and skills that adolescents gain from multicultural socialization opportunities may not directly inform their educational aspirations and expectations while academic socialization across multiple settings, or efforts to prepare youth to attend and thrive in educational settings, often emerges as an important resource in raising youth’s educational aspirations and expectations (Chun & Devall, 2019). Future work should continue to examine how different types of socialization including multicultural and academic socialization inform adolescent beliefs in their ability to realize their educational aspirations and, eventually, reach their educational goals.

In sum, schools, peers, and families are salient proximal developmental contexts comprising adolescent multicultural socialization niches. These contexts work in tandem with one another, and their joint forces may promote or inhibit multicultural socialization goals and associated academic-related benefits. Adolescents negotiating more cohesive niches with higher degrees of multicultural socialization seem to reap the most benefits, specifically they demonstrated higher behavioral and emotional academic engagement. Conversely, there was partial evidence that adolescents negotiating dissimilar niches with lower degrees of multicultural socialization seem to reap the least benefits for their academic functioning. Overall, findings underscore the importance of both consistency and degree of multicultural socialization experiences and suggest that these benefits do not extend to all indicators of academic functioning.

### Social Position Indicators of Multicultural Socialization Niches (Exploratory, Aim 3)

Based upon extant theory recognizing the influence of salient social position indicators (e.g., gender, ethnicity/race, and parental nativity; García Coll et al., 1996) on youth socialization processes, exploratory analyses examined whether these key indicators of social position shape the multicultural socialization niches youth were negotiating while accounting for school site. Findings indicated social position informed the degree and consistency of multicultural socialization opportunities that youth experience across these settings. Of note, school site was not a significant predictor with two exceptions: compared to School 4, adolescents attending School 2 were less likely to negotiate *cross-setting dissimilar peer contrast* and *cross-setting similar lower socialization* niches than the *cross-setting similar higher socialization* niche. Regarding gender, girls were more likely than boys to negotiate the *cross-setting similar moderate socialization* niche compared to most niches. This finding may suggest that girls are more likely than boys to receive consistent and frequent multicultural socialization messages across schools, peers, and families. This finding stands in contrast with prior work that has documented that gender does not inform the types (e.g., degree and consistency) of cultural socialization niches that adolescents negotiate (heritage, national, combination of cultural socialization; Byrd & Ahn, 2020; Wang & Benner, 2016). The current study’s findings, which focuses on multicultural socialization niches across three settings, are consistent with other work suggesting that girls are more likely than boys to seek cultural socialization experiences (Huynh & Fuligni, 2008) and indicating that girls are often considered the carriers of culture (Umaña-Taylor et al., 2009). Thus, girls may seek out multicultural socialization experiences across settings because they have a greater awareness of cultural influences and diversity.

Findings indicated that ethnicity/race also informed the multicultural socialization niches that adolescents negotiate on a regular basis. Specifically, Latinx and Multiethnic youth shared the same multicultural socialization niches as White youth. However, it is important to note that most Multiethnic youth (67%) identified Hispanic/Latinx as one of their ethnic-racial identities. Further, the Latinx population is the second largest ethnic-racial group (White is the largest group) in the Southwest city where the study took place. Therefore, the sample composition combined with the establishment of the Latinx population in this region of the country may explain the similarities found across Latinx and Multiethnic youth compared to White youth. Further, prior research has documented that Latinx parents highly endorse socialization values of diversity, such as talking to their children about cultural differences (Ayon, 2018), these values likely inform the multicultural socialization opportunities afforded to youth within the family setting which intersects with other settings.

No differences were found across niches for Black youth compared to White youth. These findings are consistent with prior work on heritage and national cultural socialization niches (Wang & Benner, 2016). However, they differ from prior research documenting that Black adolescents were less likely than White adolescents to negotiate a cross-setting dissimilar socialization niche involving a combination of cultural socialization opportunities and experiences of discrimination (Byrd & Ahn, 2020). Given this prior study focused on socialization and discrimination, their findings are in line with theoretical work documenting the pervasive impact of exposure to discrimination and colorism for Black youth (e.g., García Coll et al., 1996) and research highlighting the importance of ethnic-racial socialization that involves coping with discrimination (Anderson & Stevenson, 2019). Future work should continue to examine the role of ethnicity/race in different types of cultural socialization.

Regarding parental nativity, findings indicated that adolescents with at least one immigrant parent were less likely than youth with no immigrant parents to negotiate cross-setting dissimilar niches than cross-setting similar niches. Thus, these findings suggest that youth with immigrant parents may be more likely to develop in niches in which schools, peers, and families have more alignment in values and goals related to multiculturalism. Adolescents developing in families with immigrant parents, who have a closer generational connection to their heritage culture due to their family’s relatively more recent immigration, experience different affordances and demands (e.g., serving as language brokers or providing host country culture socialization) that inform their multicultural socialization experiences within the family setting (Safa et al., 2022) and beyond (Safa et al., 2019). These adolescents may seek out multicultural socialization experiences across settings because they have greater awareness of cultural affordances and demands. Prior work, however, has documented that parental nativity informed the types of national culture socialization niches that youth navigate but did not inform their heritage culture socialization niches. Specifically, adolescents with at least one immigrant parent were more likely to be in the cross-setting dissimilar national cultural socialization niche than those without immigrant parents (Wang & Benner, 2016). Findings from prior work and the current study suggest that parental nativity may differentially inform multiple cultural socialization opportunities. More work is needed in this area.

In sum, indicators of social position shape the multicultural socialization niches that adolescents navigate. Findings indicated that girls and youth with immigrant parents were more likely to negotiate more cohesive niches with relatively higher degrees of multicultural socialization opportunities than their counterparts. Further, Latinx and Multiethnic adolescents were more likely to negotiate the same niches than White youth, but no differences were observed between Black and White youth. Taken together, these findings exemplify the role of the affordances and demands youth experience based on their social position and underscore the importance of examining how intersectional identities may relate to multicultural socialization niches (Priest et al., 2014).

### Developmental and Applied Implications

The study findings have important theoretical, translational, and practical implications. Building on ecological models (e.g., García Coll et al., 1996), the current study highlights the transactional nature of youth development and adjustment by providing evidence that indicators of social position can shape youth’s context of development and that contextual diversity in multicultural socialization experiences can inform adolescent academic functioning. Furthermore, this study provides evidence of youth’s challenges in culturally diverse societies where multiculturalism values have not been widely adopted (Berry et al., 2022). Indeed, most adolescents in the current sample were negotiating niches in which they were afforded inconsistent and/or lower multicultural socialization opportunities suggesting that most adolescents have not received enough opportunities to develop multicultural competencies across three of their main proximal contexts of development.

Albeit in a small proportion of the sample, the importance of consistency and at least moderate degrees of multicultural socialization was also evident. These findings point to potential intervention targets to enhance youth’s academic functioning in ethno-racially diverse societies. Recently, promotion efforts to provide youth with opportunities to learn about their own and other’s ethnic-racial groups and cultural heritages have emerged (e.g., Dziedziewicz et al., 2014; Stein et al., 2021; Umaña-Taylor et al., 2018), but these efforts are often focused on increasing socialization opportunities in one particular context like school (e.g., Umaña-Taylor et al., 2018) or family (e.g., Stein et al., 2021). The study’s results suggest that such programs may be most effective when multiple socializing contexts are involved. Indeed, the *cross-setting dissimilar school contrast* and *peer contrast socialization* niches were associated with lower emotional or behavioral academic engagement. This suggests that youth might need additional support to make sense of the fragmented and inconsistent messages they receive in their schools, peers, and families. Thus, given the need for successful navigation of increasingly ethno-racially diverse school contexts (Nishina et al., 2019), these findings point to the meaningful role of engaging with multiple socialization contexts to promote youth academic functioning, particularly emotional and behavioral academic engagement. Cohesive niches with frequent multicultural socialization experiences might promote youth’s multicultural competencies, including engagement with ethno-racially diverse peers in academic settings, with benefits for their academic functioning (Schachner et al., 2016; Schachner et al., 2021). Future research on promoting multicultural competence should consider the relative degree and consistency of youth multicultural socialization experiences between school and other key proximal contexts (Barrett, 2018; Dee & Penner, 2017).

### Limitations and Future Directions

The use of cross-setting, person-centered analyses in a relatively large racially and ethnically diverse sample of early and middle adolescents across four schools is a strength of the study. Despite this strength, several limitations should be noted. This is a cross-sectional study, and thus changes in multicultural socialization niches could not be examined. Future work should rely on longitudinal designs and investigate cross-setting changes in the degree and consistency of multicultural socialization and how changes (or maintenance) throughout adolescence may prospectively inform academic functioning. Additionally, the bidirectional nature between cross-setting multicultural socialization and youth’s socialization seeking efforts was not captured within the current study. Consistent with prior work highlighting the role of youth agency in cultural socialization processes (Umaña-Taylor et al., 2013), it would be important for future work to examine how youth’s efforts to learn about ethnic/racial and cultural heritages other than their own shape youth’s socialization niches and their academic-related benefits.

Furthermore, for a more comprehensive understanding of cross-setting multicultural socialization niches and their academic-related associations, future research should rely on multi-reporter assessments (e.g., parent, youth), as well as multi-method approaches such as surveys and observations of multicultural socialization in families, peers, teachers, and schools and the use of school records as additional indicators of academic functioning. Relatedly, the school multicultural socialization scale used in this study assessed the degree to which youth agree with statements about cultural pluralism to be true or not, whereas the friends/peers and the parent/caregiver scale assessed how frequent opportunities to learn about cultural pluralism and equal treatment for members of all groups were available to them. It is possible that the scale and content of the items limited the variability that emerged in school multicultural socialization levels across the identified niches. Thus, future work should measure multicultural socialization efforts relative to cultural pluralism and equal treatment across settings.

Given the nature of the study’s sample, the present findings may not generalize to youth from other ethnic-racial groups and youth attending schools with differing ethnic-racial compositions across various U.S. regions. Although this study tested whether the youth’s ethnic-racial background (i.e., White, Black, Latinx, Multiethnic) predicted the likelihood to be in a specific multicultural socialization niche, youth who identified as Asian American or Pacific Islander, American Indian or Alaska Native, or Arab, Middle Eastern, or North African (*n* = 27) were omitted from this analysis due to the small sample size. This study did not examine whether the identified niches were comparable (i.e., invariant) across ethnicity/race because of insufficient sample size in each ethnic-racial group to conduct such analyses (Morin et al., 2015). Future work should recruit larger multigroup samples to understand better the links between ethnic-racial backgrounds and multicultural socialization niches. Relatedly, this study tested the role of key social position indicators (i.e., ethnicity-race, gender, parent nativity) on profile membership but due to the analytical approach used, this study could not test the role of these factors on the association between multicultural socialization niches and indicators of academic functioning. This is an important future direction.

While the identification of the niches was justified by the data and surfaced conceptually meaningful groupings, some of these niches or profile sizes were comparatively small, particularly the *cross-setting dissimilar greater peer contrast socialization* niche and the *cross-setting similar higher socialization* niche. Identifying these groups is meaningful and important, but caution is warranted in interpreting group comparisons involving these niches. Specifically, the small size of the groups may lead to comparisons with lower statistical power.

It is also possible that the study’s data collection period (December – January) had some sway on opportunities to discuss and learn about different ethnic-racial and cultural heritages, given that multiple holidays are celebrated across cultures during that time; data collection at several periods during the year might yield insights in temporal dynamics of multicultural socialization across settings. Finally, possible mediating associations were not tested. For instance, given its aim to teach intercultural competence and understanding, multicultural socialization might promote critical thinking skills closely linked to academic functioning (Tadmor et al., 2009). Future research might formally test this possibility.

## Conclusion

Cultural diversity has shaped many parts of the world and has important implications for the development and adjustment of youth from all ethnic and racial groups, particularly for their academic adjustment as youth increasingly attend ethno-racially diverse schools. Nonetheless, there is scarce research on how socialization processes prepare youth to respond to increasing multicultural demands and the degree to which these socialization opportunities inform youth academic functioning. This study addressed this gap by examining multicultural socialization niches across key proximal settings (i.e., schools, peers, and families) and their links with youth academic functioning. Findings from the current study highlight that U.S. adolescents from multiple ethno-racial backgrounds are negotiating a diverse range of multicultural socialization niches that vary in the degree and consistency in socialization experiences across school, peer, and family settings: *cross-setting similar higher, moderate*, *and lower socialization niches and cross-setting dissimilar peer contrast*, *greater peer contrast*, *and school contrast socialization niches*. Further, the settings comprising these niches work in tandem with one another, and their joint forces inform multicultural socialization goals and associated academic-related benefits. Particularly, adolescents negotiating more cohesive niches with higher degrees of multicultural socialization demonstrated higher behavioral and emotional academic engagement. Conversely, there was partial evidence that adolescents negotiating dissimilar niches with lower degrees of multicultural socialization demonstrated lower academic functioning. In addition, findings from exploratory analyses indicated that social position could shape the multicultural socialization opportunities that youth experience across these settings. Girls and youth with at least one immigrant parent were more likely to negotiate cohesive niches with higher degrees of multicultural socialization compared to their counterparts. Further, Latinx and Multiethnic youth were more likely to negotiate the same niches than White youth, but no differences were observed between Black and White youth. Study findings highlight the transactional nature of youth development and adjustment by providing evidence that social position informs youth’s context of development and that contextual diversity in multicultural socialization experiences informs their academic functioning. Importantly, promoting multicultural socialization across school, peer, and family settings is promising for improving youth’s academic functioning.

## Supplementary Information

Supplementary Information
