# Supplementary material for: A Person-Centered Analysis of Adolescent Multicultural Socialization Niches and Academic Functioning
Source: J Youth Adolesc. 2023 Jul 26;52(11):2261–84. doi: 10.1007/s10964-023-01828-0 (PMC10495488; doi:10.1007/s10964-023-01828-0)
Supplement: Supplementary file 1 — Supplementary Information [file 10964_2023_1828_MOESM1_ESM.docx]

**Appendix A**

Based on several statistical fit indices reported in the manuscript, the 6-profile solution was optimal for describing patterns of multicultural socialization niches across school, peers, and family settings. However, the 5-profile solution had many similar niches. Thus, we also present the findings on the relations between multicultural socialization niches and academic functioning (Table A1), associations between social position indicators and latent profile membership (Table A2), and a figure of multicultural socialization niches based on the 5-profile solution (Figure A1).

**Table A1**

*Aim 2: Associations Between the 5-Profile LPA Solution of Multicultural Socialization Niches and Academic Functioning*

|  | Emotional Academic Engagement  *(n = 701)* | | Behavioral  Academic  Engagement  *(n = 702)* | | Academic  Aspirations  *(n = 683)* | | Academic  Expectations  *(n = 682)* | |  |
| --- | --- | --- | --- | --- | --- | --- | --- | --- | --- |
| **Academic Functioning Means by Niche** | *M* | *SE* | *M* | *SE* | *M* | *SE* | *M* | *SE* |  |
| 1. Cross-setting dissimilar peer contrast socialization niche | 2.169 | 0.061 | 2.768 | 0.064 | 5.228 | 0.211 | 4.566 | 0.131 |  |
| 2. Cross-setting dissimilar greater peer contrast socialization niche | 2.565 | 0.241 | 3.261 | 0.310 | 5.261 | 0.629 | 4.881 | 0.332 |  |
| 3. Cross-setting dissimilar school contrast socialization niche | 2.134 | 0.094 | 2.756 | 0.091 | 4.915 | 0.210 | 3.991 | 0.161 |  |
| 4. Cross-setting similar lower socialization niche | 2.447 | 0.076 | 2.893 | 0.077 | 5.500 | 0.259 | 4.523 | 0.160 |  |
| 5. Cross-setting similar higher socialization niche | 3.027 | 0.131 | 3.400 | 0.070 | 5.326 | 0.216 | 5.064 | 0.288 |  |
|  |  |  |  |  |  |  |  |  |  |
| **Wald Tests Comparisons Across Niches** | χ ^2^ (1) = | *d* | χ ^2^ (1) = | *d* | χ ^2^ (1) = | *d* | χ ^2^ (1) = | *d* |  |
| *5. Cross-setting similar higher socialization niche vs.*: |  |  |  |  |  |  |  |  |  |
| 1. Cross-setting dissimilar peer contrast socialization niche | **35.083^***^** | **0.820** | **44.190^***^** | **0.585** | 0.119 | 0.028 | 2.478 | 0.221 |  |
| 2. Cross-setting dissimilar greater peer contrast socialization niche | 2.829 ^†^ | 0.339 | 0.190 | 0.083 | 0.009 | 0.019 | 0.172 | 0.090 |  |
| 3. Cross-setting dissimilar school contrast socialization niche | **30.689^***^** | **0.826** | **31.502^***^** | **0.633** | 1.811 | 0.173 | **10.576^**^** | **0.566** |  |
| 4. Cross-setting similar lower socialization niche | **13.737^***^** | **0.607** | **22.960^***^** | **0.541** | 0.228 | -0.055 | 2.497 | 0.268 |  |
| *4. Cross-setting similar lower socialization niche vs.:* |  |  |  |  |  |  |  |  |  |
| 1. Cross-setting dissimilar peer contrast socialization niche | **6.623^*^** | **0.266** | 1.112 | 0.115 | 0.385 | 0.076 | 0.035 | -0.019 |  |
| 2. Cross-setting dissimilar greater peer contrast socialization niche | 0.222 | -0.101 | 1.338 | -0.277 | 0.143 | 0.066 | 0.965 | -0.168 |  |
| 3. Cross-setting dissimilar school contrast socialization niche | **6.831^**^** | **0.295** | 1.520 | 0.130 | **3.816^*^** | **0.194** | **5.717^*^** | **0.263** |  |
| *3. Cross-setting dissimilar school contrast socialization niche vs.:* |  |  |  |  |  |  |  |  |  |
| 1. Cross-setting dissimilar peer contrast socialization niche | 0.090 | -0.032 | 0.009 | -0.011 | 0.912 | -0.093 | **6.581^*^** | **-0.262** |  |
| 2. Cross-setting dissimilar greater peer contrast socialization niche | 2.265 | -0.336 | 1.936 | -0.356 | 0.216 | -0.113 | **4.956^*^** | **-0.437** |  |
| *2. Cross-setting dissimilar greater peer contrast socialization niche vs.:* |  |  |  |  |  |  |  |  |  |
| 1. Cross-setting dissimilar peer contrast socialization niche | 2.306 | 0.340 | 2.164 | 0.379 | 0.002 | 0.009 | 0.705 | 0.137 |  |
|  |  |  |  |  |  |  |  |  |  |

*Note*. *Note*. *M* = Mean; *SE* = Standard Error; VS.= Versus. **Boldface** represents significant Wald tests (*p* < .05) indicating mean level differences across compared niches.

**Table A2**

*Exploratory Aim 3: Multinomial Logistic Regression Analyses: Associations Between Social Position and the 5-Profile LPA Solution of Multicultural Socialization Niches (n = 659)*

|  | Niches 1 vs. 2 | | | Niches 1 vs. 3 | | | Niches 1 vs. 4 | | | Niches 1 vs. 5 | | | Niches 2 vs. 3 | | |
| --- | --- | --- | --- | --- | --- | --- | --- | --- | --- | --- | --- | --- | --- | --- | --- |
|  | Coef. | SE | OR | Coef. | SE | OR | Coef. | SE | OR | Coef. | SE | OR | Coef. | SE | OR |
| Girl ^a^ | -0.117 | 0.479 | 0.890 | 0.109 | 0.284 | 1.115 | -0.479^†^ | 0.283 | 0.619 | 0.480 | 0.471 | 1.616 | 0.226 | 0.530 | 1.254 |
| Immigrant parent/s ^b^ | -0.131 | 0.522 | 0.877 | -0.089 | 0.359 | 0.915 | **-0.687^*^** | **0.319** | **0.503** | **-1.155^*^** | **0.481** | **0.315** | 0.042 | 0.570 | 1.043 |
| Latinx | -0.389 | 0.662 | 0.678 | 0.382 | 0.395 | 1.465 | -0.205 | 0.426 | 0.815 | **1.317^*^** | **0.563** | **3.732** | 0.770 | 0.692 | 2.160 |
| Black | -0.833 | 0.846 | 0.435 | 0.468 | 0.713 | 1.597 | -0.474 | 0.654 | 0.623 | 0.228 | 0.789 | 1.256 | 1.301 | 0.992 | 3.673 |
| Multiethnic | 0.236 | 0.660 | 1.266 | 0.215 | 0.356 | 1.240 | -0.338 | 0.401 | 0.713 | **1.837^*^** | **0.768** | **6.278** | -0.021 | 0.703 | 0.979 |
| White ^c^ | **--** | **--** | **--** | **--** | **--** | **--** | **--** | **--** | **--** | **--** | **--** | **--** | **--** | **--** | **--** |
| School 1 | -0.492 | 0.517 | 0.611 | -0.300 | 0.335 | 0.741 | 0.000 | 0.346 | 1.000 | -0.267 | 0.599 | 0.766 | 0.192 | 0.555 | 1.212 |
| School 2 | 0.850 | 1.775 | 2.340 | -0.821 | 0.557 | 0.440 | -0.373 | 0.530 | 0.689 | **-1.614^*^** | **0.686** | **0.199** | -1.671 | 1.810 | 0.188 |
| School 3 | 0.012 | 0.647 | 1.012 | 0.148 | 0.399 | 1.160 | -0.031 | 0.402 | 0.969 | -0.235 | 0.569 | 0.791 | 0.136 | 0.692 | 1.146 |
| School 4 ^d^ | **--** | **--** | **--** | **--** | **--** | **--** | **--** | **--** | **--** | **--** | **--** | **--** | **--** | **--** | **--** |
|  |  |  |  |  |  |  |  |  |  |  |  |  |  |  |  |
|  | Niches 2 vs. 4 | | | Niches 2 vs. 5 | | | Niches 3 vs. 4 | | | Niches 3 vs. 5 | | | Niches 4 vs. 5 | | |
|  | Coef. | SE | OR | Coef. | SE | OR | Coef. | SE | OR | Coef. | SE | OR | Coef. | SE | OR |
| Girl ^a^ | -0.362 | 0.475 | 0.696 | 0.597 | 0.617 | 1.817 | **-0.588^*^** | **0.291** | **0.555** | 0.371 | 0.488 | 1.449 | 0.959^†^ | 0.506 | 2.609 |
| Immigrant parent/s ^b^ | -0.556 | 0.493 | 0.573 | -1.024 | 0.634 | 0.359 | -0.598^†^ | 0.326 | 0.550 | **-1.066^*^** | **0.497** | **0.344** | -0.468 | 0.503 | 0.626 |
| Latinx | 0.183 | 0.655 | 1.201 | **1.705^*^** | **0.777** | **5.501** | -0.587 | 0.413 | 0.556 | 0.935 | 0.579 | 2.547 | **1.522^*^** | **0.614** | **4.581** |
| Black | 0.359 | 0.831 | 1.432 | 1.061 | 0.975 | 2.889 | -0.942 | 0.683 | 0.390 | -0.240 | 0.821 | 0.787 | 0.702 | 0.807 | 2.018 |
| Multiethnic | -0.573 | 0.672 | 0.564 | 1.601^†^ | 0.964 | 4.958 | -0.553 | 0.393 | 0.575 | **1.622^*^** | **0.778** | **5.063** | **2.175^**^** | **0.824** | **8.802** |
| White ^c^ | **--** | **--** | **--** | **--** | **--** | **--** | **--** | **--** | **--** | **--** | **--** | **--** | **--** | **--** | **--** |
| School 1 | 0.491 | 0.502 | 1.634 | 0.225 | 0.712 | 1.252 | 0.299 | 0.347 | 1.349 | 0.033 | 0.609 | 1.034 | -0.266 | 0.632 | 0.766 |
| School 2 | -1.223 | 1.714 | 0.294 | -2.464 | 1.800 | 0.085 | 0.448 | 0.468 | 1.565 | -0.793 | 0.676 | 0.452 | -1.241^†^ | 0.690 | 0.289 |
| School 3 | -0.042 | 0.641 | 0.959 | -0.246 | 0.779 | 0.782 | -0.178 | 0.421 | 0.837 | -0.383 | 0.608 | 0.682 | -0.204 | 0.626 | 0.815 |
| School 4 ^d^ | **--** | **--** | **--** | **--** | **--** | **--** | **--** | **--** | **--** | **--** | **--** | **--** | **--** | **--** | **--** |
|  |  |  |  |  |  |  |  |  |  |  |  |  |  |  |  |

*Note*. VS.= Versus; Coef. = Coefficient; *SE* = standard error; *OR* = odds ratio. **Boldface** represents significant estimates (*p* < .05) indicating that a given social position indicator was a significant predictor of profile membership across compared niches, specifically estimates reflect the effects of the predictors on the likelihood of membership into the first versus second listed niche. Niche 1: Cross-setting dissimilar peer contrast socialization; Niche 2: Cross-setting dissimilar greater peer contrast socialization; Niche 3: Cross-setting dissimilar school contrast socialization; Niche 4: Cross-setting similar lower socialization; and Niche 5: Cross-setting similar higher socialization.

^a^ Girl (1 = *girl*; 0 = *boy*)

^b^ Immigrant parent/s (1 = *at least one parent born abroad*; 0 = *both parents born in the U.S.*)

^c^ White was coded as the reference group across model comparisons examining the role of ethnicity/race.

^d^ School 4 (largest sample size) was coded as the reference group across model comparisons and treated as a control variable to account for the nested structure of data.

^†^ *p* < .10, ^*^ *p* < .05, ^**^ *p* < .01, ^***^ *p* < .001.

**Figure A1**

*Aim 1: Means and Proportions for the 5-Profile LPA Solution of Multicultural Socialization Niches (n = 704)*

*Note*. Niche 1: Cross-setting dissimilar peer contrast socialization niche (*n* = 309); Niche 2: Cross-setting dissimilar greater peer contrast socialization niche (*n* = 47); Niche 3: Cross-setting dissimilar school contrast socialization niche (*n* = 147); Niche 4: Cross-setting similar lower socialization niche (*n* = 170); and Niche 5: Cross-setting similar higher socialization niche (*n* = 31). Average multicultural socialization scores are depicted under niche labels. Final counts for the latent profiles are based on classified profile membership.

**Appendix B**

**Table B**

*Most Likely Profile Membership from the 6-Profile LPA Solution of Multicultural Socialization Niches by Social Position Indicators*

|  | | Niche 1: Cross-setting dissimilar peer contrast socialization | Niche 2: Cross-setting dissimilar greater peer contrast socialization | Niche 3: Cross-setting dissimilar school contrast socialization | Niche 4: Cross-setting similar lower socialization | Niche 5: Cross-setting similar moderate socialization | Niche 6: Cross-setting similar higher socialization |
| --- | --- | --- | --- | --- | --- | --- | --- |
|  | |  |  |  |  |  |  |
| *Profile size:* | | *n* = 286 | *n* = 41 | *n* = 119 | *n* = 177 | *n* = 52 | *n* = 29 |
|  |  |  |  |  |  |  |  |
|  |  |  |  |  |  |  |  |
| **Social Position Indicators** | *% of sample* |  |  |  |  |  |  |
| ***Gender*** |  |  |  |  |  |  |  |
| Girl | 50% | 51% | 49% | 46% | 49% | 71% | 35% |
| Boy | 49% | 49% | 51% | 53% | 49% | 27% | 62% |
|  |  |  |  |  |  |  |  |
| ***Parental nativity*** |  |  |  |  |  |  |  |
| At least one parent born abroad | 33% | 28% | 37% | 31% | 36% | 56% | 48% |
| Both parents born in the United States | 67% | 73% | 63% | 69% | 64% | 44% | 52% |
|  |  |  |  |  |  |  |  |
| ***Ethnicity/Race*** |  |  |  |  |  |  |  |
| Latinx | 32% | 31% | 44% | 28% | 32% | 44% | 27% |
| White | 26% | 27% | 20% | 30% | 24% | 12% | 41% |
| Black | 7% | 6% | 12% | 6% | 9% | 4% | 14% |
| Multiethnic | 32% | 32% | 24% | 33% | 32% | 39% | 10% |
| AAPI ^a^, AI/AN ^b^, or AMENA ^c^ | 4% | 4% | 0% | 3% | 5% | 2% | 7% |
|  |  |  |  |  |  |  |  |

*Note*. Percentages across social position indicators within the sample and each most likely profile are rounded to whole percentage points and might not equal 100% due to rounding or missing data. Profile size is based on the estimated model’s probabilistic likelihood of profile membership.

^a^ AAPI (*Asian American or Pacific Islander*).

^b^ AI/AN (*American Indian or Alaska Native*).

^c^ AMENA (*Arab, Middle Eastern, or North African*).
